# The Impact on Survival of Neoadjuvant Treatment Interruptions in Locally Advanced Rectal Cancer Patients

**DOI:** 10.3390/jpm14030266

**Published:** 2024-02-29

**Authors:** Horia-Dan Lișcu, Ionut-Lucian Antone-Iordache, Dimitrie-Ionuț Atasiei, Ioana Valentina Anghel, Andreea-Teodora Ilie, Taraneh Emamgholivand, Andreea-Iuliana Ionescu, Florica Șandru, Christopher Pavel, Flavia Ultimescu

**Affiliations:** 1 Discipline of Oncological Radiotherapy and Medical Imaging, “Carol Davila” University of Medicine and Pharmacy, 050474 Bucharest, Romania; horia-dan.liscu@drd.umfcd.ro (H.-D.L.); ionut.atasiei@stud.umfcd.ro (D.-I.A.); ioana.anghel@stud.umfcd.ro (I.V.A.); teodora.i.ilie@stud.umfcd.ro (A.-T.I.); emamgholivand.taraneh@stud.umfcd.ro (T.E.); andreea-iuliana.miron@drd.umfcd.ro (A.-I.I.); 2 Radiotherapy Department, Colțea Clinical Hospital, 030167 Bucharest, Romania; 3 Medical Oncology Department, Colțea Clinical Hospital, 030167 Bucharest, Romania; 4 Department of Dermatology, Elias University Emergency Hospital, 011461 Bucharest, Romania; florica.sandru@umfcd.ro; 5 Department of Gastroenterology, “Carol Davila” University of Medicine and Pharmacy, 050474 Bucharest, Romania; christopher-jesse-vlad.pavel@drd.umfcd.ro; 6 Department of Pathology, Institute of Oncology “Prof. Dr. Alexandru Trestioreanu”, 022328 Bucharest, Romania; flavia.ultimescu@drd.umfcd.ro; 7 Department of Pathology, “Carol Davila” University of Medicine and Pharmacy, 020021 Bucharest, Romania

**Keywords:** neoadjuvant radiotherapy, total neoadjuvant treatment, locally advanced rectal cancer, downstage, treatment interruptions

## Abstract

The standard oncologic treatment of locally advanced rectal cancer is long-course radio-chemotherapy followed by surgery and adjuvant chemotherapy. This can result in a lengthy total treatment duration, sometimes up to one year from the diagnosis. Interruptions to neoadjuvant treatment can occur for a variety of reasons, forced or unforced. The main purpose of this study is to analyze the survival data of locally advanced rectal cancer patients who received neoadjuvant treatment and to find a cut-off point showing exactly how many days of interruption of neoadjuvant treatment the risk of death or disease relapse increases. We conducted a retrospective study on 299 patients with locally advanced rectal cancer using survival analysis (Kaplan–Meier curve and Cox regression) to determine survival probabilities for overall survival, local control, and disease-free survival. Patients with 0 to 3 days of neoadjuvant therapy interruption had a higher overall survival probability compared to patients with 4 or more days (90.2% compared to 57.9%, *p*-value < 0.001), hazard ratio 5.89 (*p* < 0.001). Local control and disease-free survival had a higher probability in patients with 0–2 days of interruption compared to people with 3 or more days (94% vs. 75.4%, and 82.2% vs. 50.5%, respectively, both *p*-values < 0.001). Patients with tumoral or nodal downstaging experienced fewer days of interruption than patients with no downstage. These findings reinforce the need for radiation oncologists to be well-organized when starting neoadjuvant treatment for rectal cancer, in order to anticipate and prevent potential treatment interruptions and achieve the best therapeutic results.

## 1. Introduction

Multimodality treatment of locally advanced rectal cancer (LARC) has been the subject of several changes in recent decades, with one of the additions being the concept of neoadjuvant treatment with radiotherapy and chemotherapy before surgery. These changes have improved the local control (LC) and survival of patients with LARC [1]. While adjuvant chemotherapy has a well-established role in colorectal cancer, some debate regarding its use in the early stages of rectal cancer still exists [2,3,4,5]. Surgical intervention has been considered the cornerstone in the curative treatment of rectal cancer for decades and has seen significantly better results after the definition of total mesorectal excision (TME) and its implementation as a surgical rule [6,7], which might change in the future, depending on the results of clinical trials proposing organ preservation [8,9,10,11].

The neoadjuvant treatment for LARC most commonly used in the US and Western Europe is called “long course radio-chemotherapy” (LCRT) and consists of radiotherapy with conventional doses (180–200 cGrays), concurrent with daily antimetabolite chemotherapy: 5-Fluorouracil or the prodrug Capecitabine. The total doses used in conventional radiotherapy for rectal cancer range from 45 Grays to 60 Grays administered over 25–30 sessions. However, there is no evidence that higher doses (54–60 Gy) necessarily bring overall survival (OS) benefits compared to standard doses (45–50 Gy) [12]. The addition of chemotherapy to neoadjuvant radiotherapy has a radiosensitizing role and has favorable effects in terms of local relapse, without significant differences in overall survival [13]. Another radiotherapy regimen, usually administered alone, is the “short course radiotherapy” (SCRT). This approach is preferred in Northern Europe and involves hypofractionation, with 25 Grays administered in 5 fractions. Total neoadjuvant treatment (TNT) is a relatively new therapeutic regimen used in the treatment of LARC, which involves the administration of maximal oncological treatment before radical surgery with improved results, especially in terms of disease-free survival (DFS) [14,15,16].

However, current treatment for LARC requires a minimum of 3 curative cancer therapies (radiotherapy, chemotherapy, surgery) administered in varying sequences and with a very long total treatment duration (TTD), which can extend up to almost a year from the time of diagnosis, depending on the stage of the disease. The TTD impacts the patient on all levels (socially, financially, emotionally, and quality of life), which can lead to forced or unforced interruptions of the treatment. These can be caused both by the nature of the disease or by the treatment: the evolution of the disease, toxicities or complications of treatment, associated comorbidities that may worsen, psychological aspects and low compliance represent several causes of therapy interruptions. Not to be forgotten is the aspect of health infrastructure that can impact the TTD, through problems of uptime of equipment in the case of radiotherapy departments, problems of supply of various chemotherapy drugs, or of timely scheduling of surgery. A recent example that had a major impact on the organization of healthcare services was the COVID-19 pandemic, whose negative oncological consequences are still being felt worldwide [17,18,19,20]. Currently, there are some strategies to compensate for the lost radiation dose, but the precise calculation of this dose has limitations and the recovery in absolute dose does not always translate into clinical results. In some situations, it is impossible to intervene with compensation strategies due to patient decisions, toxicities, physician decisions, or health infrastructure, and for these patients, the impact of prolonging the TTD is not very clear. This is the main reason why we conducted this study, to give us a clearer picture of the impact of interruption to neoadjuvant treatment.

The main objective of this study is to provide an overview of the negative impact of LCRT interruptions and their influence on OS, LC, and DFS. We aimed to identify the most sensitive and specific cut-off point, measured in days of interruptions of neoadjuvant treatment, dividing our group of patients into one with a lower risk and one with a higher risk of occurrence of the undesirable event (death, local relapse, or relapse of the disease). Simply put, we aimed to find exactly how many days of interruption the risk of suffering an outcome starts to increase.

As a secondary objective, we evaluated the days of treatment interruption and their influence on tumor and nodal downstaging after neoadjuvant therapy, knowing that downstaging has been proposed as a predictive factor for the outcome of patients with rectal cancer [21,22,23].

## 2. Materials and Methods

### 2.1. Study Population

We conducted a retrospective, multi-institutional study of 299 patients with LARC stages II–III at diagnosis. Patients who were admitted to Colțea Clinical Hospital in Bucharest between 1 January 2004 and 31 December 2020 with a diagnosis of advanced local rectal neoplasm and who had undergone at least 1 of the 3 oncological treatments commonly used (radiotherapy, chemotherapy, or surgery) in this hospital were included in the study if they met the inclusion criteria:

- Patient with clinical stage II–III rectal neoplasm;
- No distant metastases at the time of diagnosis;
- ECOG score between 0 and 2;
- Receiving neoadjuvant radiotherapy or radio-chemotherapy with total doses between 45 and 50.4 Gray;
- Surgery with radical intent.

Patients staged T1-2N0 were excluded. Patients with poor performance status, ECOG > 2, or who received neoadjuvant external radiotherapy with doses outside the range chosen as inclusion criteria (patients who did not complete radiotherapy, who were short-course-irradiated or who received extra dose compensation for days of interruption) were excluded as well.

Imaging data at diagnosis for clinical staging (pelvis MRI, chest CT scan, and colonoscopy), postoperative histopathological data (histopathological bulletin with pathological staging), and local or distant survival and recurrence data were collected by trained investigators retrospectively by consulting medical records from post-treatment follow-ups and by directly contacting either the patient or their legal representative. All patients were followed up after treatment at 3–6 months in the first 2 years and then at 6–12 months according to institutional protocols.

### 2.2. Treatment and Follow-Up

All patients received external rectal and pelvic radiotherapy with total doses ranging from 45 to 50.4 Gy. External radiotherapy was delivered with a linear accelerator (LINAC) using 6 MV photons by 2D-conformational, 3D-conformational (3D-CRT), or intensity-modulated techniques, either intensity-modulated radiotherapy (IMRT) or volumetric-modulated arc therapy (VMAT), depending on the radiation oncologist’s decision and radiotherapy clinic’s availability. The doses used per fraction were in the range of 180–200 cGy and the total number of sessions received by each patient in the study varied between 25 and 28. Patients irradiated using the 2D-conformational technique were irradiated through 4 AP PA LR RL box-type fields with conformal fields for whole pelvis irradiation. These patients were positioned at the LINAC on the tattoo points performed in the conventional simulation and were instructed to have an empty rectum and bladder before each irradiation session.

Patients irradiated with 3D-CRT, IMRT, and VMAT techniques were scanned on the CT simulator, contoured using the clinic’s Treatment Planning Software, and irradiated with prior verification of correct machine positioning using IGRT methods (minimum MV; kV or CBCT where equipment allowed). Patients irradiated using modern techniques were instructed to have a comfortably full bladder (urinate, then drink 500 mL water 30 min before scanning and sessions) and an empty rectum. The treatment plan was carried out respecting the irradiation with a minimum of 45–46 Gy of the entire mesorectum and pelvic lymph node areas: obturator nodal group, internal iliac nodal group up to the bifurcation of the common iliac vessels, and external iliac nodal group for high-risk patients. Some patients received a boost up to 50–50.4 Gy in 2–3 sessions to the rectal tumor.

Neoadjuvant treatment was supplemented with oral capecitabine at a dose of 825 mg/m^2^ twice daily in patients considered suitable for such a radiosensitizing treatment. Capecitabine was prescribed by the medical oncologist after clinical, biological, and ECOG performance status (0–1) evaluation, and according to patient comorbidities.

Surgery was scheduled 8–12 weeks after completion of neoadjuvant treatment and only patients who were proposed a curative treatment plan with TME were included. Surgical approaches were chosen by the attending surgeon according to the location of the tumor, ranging from low or ultra-low anterior resection, abdominoperineal resection, delayed colonic anastomosis, or partial mesorectal resection. Patients were monitored postoperatively, and those who received temporary colostomy were called for a second colostomy reintegration surgery 3–12 months postoperatively.

Post-treatment follow-up was performed by the treating physicians (radiation oncologist and/or medical oncologist and/or general surgeon) at regular intervals: every 3–6 months in the first 2 years and 6–12 months in years 2–5. Follow-up involved history and clinical examination. If necessary, in case of suspicion of recurrence and/or metastasis, an imaging examination was performed with pelvic MRI, chest and abdominal CT, or colonoscopy. For the purpose of this study, all patients were contacted by telephone to express their consent to participate in the study and to provide any missing medical documents for the completion of statistical data. In the case of deceased patients, the closest of kin was approached.

### 2.3. Statistical Analysis

JASP 0.18.3, SPSS 29.0.2, and R 4.3.2 were used for statistical analysis. Descriptive statistics were computed with JASP, while the other two software were used for the rest of our tests.

Nominal data are presented as frequencies (percentages) and number of patients for each category. Continuous variables (age, number of days of interruption for every reason) are presented as mean and standard deviation.

We performed Mann–Whitney independent sample U tests for continuous variables while categorical data were analyzed with contingency tables, testing for significance with chi-square.

Survival analysis was performed by generating 60-month Kaplan–Meier curves and assessing their differences by log-rank tests. The following assumptions were verified: censoring was unrelated to the outcome measured, we considered the same survival probability for people recruited early and late during our study, and the outcomes happened as close to the specified times as possible, considering follow-up.

In order to obtain a cut-off value for days of neoadjuvant therapy interruption that best discriminates among those who suffer an event (death, relapse) and those who do not, we used receiver operating characteristic curve analysis and Youden’s index. For overall survival, we applied a univariate Cox proportional hazards model to calculate the hazard ratio between the cut-off separated groups. To test the proportional hazards assumption of the Cox model, we used cox.zph function in R to generate Schoenfeld residuals. The linearity assumption is always met when predictors are binary categorical predictors. Other considered assumptions: censoring was unrelated to the outcome; distinct individuals have independent survival times.

For every test performed, a *p*-value of 0.05 or less was considered statistically significant.

This study was approved by the Ethics Committee of Colțea Clinical Hospital Bucharest according to decision number 34 from 14.12.2023.

## 3. Results

**Figure 1:**
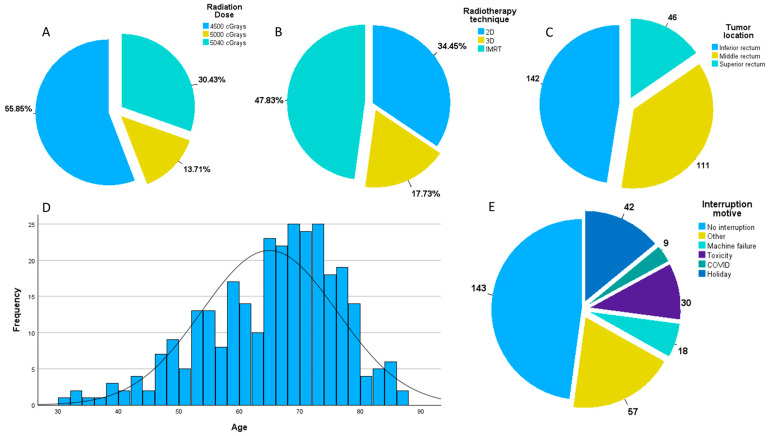
(**A**) Distribution of patients by radiation dose; (**B**) distribution of patients by radiotherapy technique; (**C**) distribution of patients by tumor location; (**D**) distribution of patients by age; (**E**) distribution of patients by interruption reason.

Our study involved 299 patients, with an average age of 64.9 years (SD 11.2), a mean neoadjuvant interruption time of 2.4 days (SD 4), and a maximum of 24 days. The rest of our population’s characteristics are presented in Table 1 and Figure 1. COVID and radiation toxicity were responsible for only 5.8% and 19.23% of interruptions (as seen in Figure 1E) with a mean of 15.9 days and 5.1 days, respectively. Although holidays and machine failure were responsible for a bigger proportion of interruptions (26.9% and 11.5%) they accounted for fewer days of interruptions (as seen in Table 1).

### 3.1. Global Survival Characteristics

**Figure 2:**
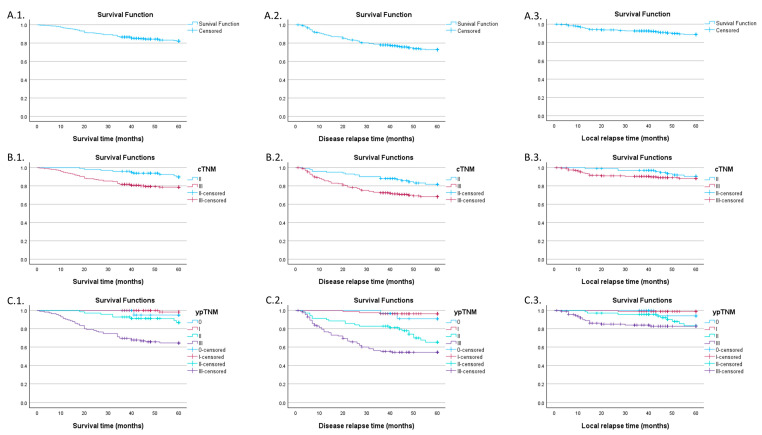
(**A.1**) Overall survival for the whole population; (**A.2**) DFS for the whole population; (**A.3**) LC for the whole population; (**B.1**) overall survival for cTNM stages II and III; (**B.2**) DFS for cTNM stages II and III; (**B.3**) local control for cTNM stages II and III; (**C.1**) overall survival by ypTNM stages; (**C.2**) DFS by ypTNM stages; (**C.3**) LC by ypTNM stages.

Kaplan–Meier survival analysis regarding overall survival, DFS, and local relapse was computed for a series of independent variables such as clinical TNM (cTNM) and post-neoadjuvant pathologic staging TNM (ypTNM) stages as well as for the whole population (Figure 2).

The 60-month survival probability was 82.3% (SE 2.3%), in terms of DFS we had a 72.8% (SE 2.7%) probability of 60 months without disease relapse, while the probability of LC was 88.8% (SE 2%). When accounting for the cTNM stage, overall survival probability was 89.7% (SE 3.3%) for stage II and 78.7% (SE 3.0%) for stage III with a statistically significant log-rank test (*p* = 0.007). The former had a DFS probability of 81.8% (SE 4.1) while the latter had only 68.3% (SE 3.5%), showing a significant difference (*p* = 0.007). For local relapse the log-rank test was not statistically significant (*p* = 0.297), with stage II and III probabilities being very close at the 60-month mark: 90.5% (SE 3.3%) and 89.1% (SE 2.3%) respectively.

When grouping by ypTNM stage, overall survival seems to be worse for stage III, with a probability of 64.5% (SE 4.5%), the difference being statistically significant (*p* < 0.001). Disease relapse happened more frequently in patients with stage II (65.3% SE 6.4% DFS probability) and stage III (54.3%, SE 4.8% DFS probability) with a significant log-rank test (*p* < 0.001). The curves seem to move closer at the 40-month mark, signifying a possible increase in relapse risk for the stage II population. The same phenomenon happens if we investigate the local relapse events; the probability of LC for stage II begins to fall at around 40 months, resulting in a 60-month probability of 83.2% (SE 5.2%), while for stage III the probability was 82.8% (SE 3.7%) with a statistically significant log-rank test when taking every stage into account (*p* = 0.001).

Additional data in Table A1.

### 3.2. Impact of Neoadjuvant Therapy Interruption Days

**Figure 3:**
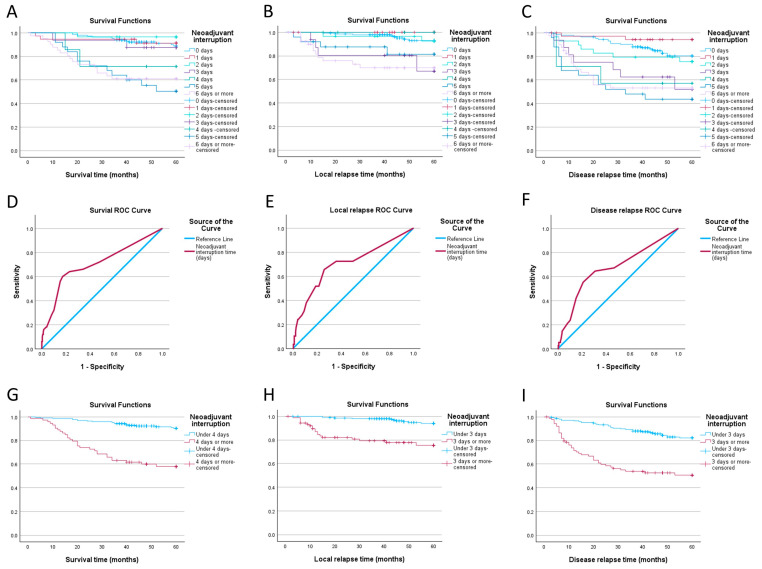
(**A**) Overall survival divided by the number of interruption days; (**B**) local control divided by number of interruption days; (**C**) disease-free survival divided by number of interruption days; (**D**) ROC curve for survival; (**E**) ROC curve for local relapse; (**F**) ROC curve for disease relapse; (**G**) overall survival divided by the 3.5 days of interruption cut-off; (**H**) disease-free survival divided by the 2.5 days of interruption cut-off; (**I**) local control divided by the 2.5 days of interruption cut-off.

In order to see after which number of days of interruption the survival probability begins to change, we first grouped the patients with the same amount of gap days and computed the Kaplan–Meier curves. As seen in Figure 3, for OS we can observe that the probability curve for patients with 4 days of interruption seems to be lower than the ones of the patients with a smaller amount of gap days (71.4%, SE 17% compared to 87.5%, SE 8.3% for 3 days). A similar phenomenon occurs for DFS and LC, as the curves for patients with a 3-day gap seem to distance from those of patients with 0, 1, and 2 days. In terms of DFS, we saw a probability of no-relapse survival of 52.1% (SE 13.9%) for 3 days compared to 75.5% (SE 8.1%) for 2 days, while the probability for LC was 67% (SE 14.9%) in the 3-day group versus 92.5% (SE 5.1%) in the 2-day group. The log-rank test *p*-value for the three curves was <0.001. More data can be found in Table A2.

Using the number of days of neoadjuvant interruption as a variable, and survival, disease relapse, and local relapse as outcomes, we built three basic risk models. In order to evaluate them and find a cut-off point that best discriminates between events we used receiver operating characteristic analysis and computed 3 curves seen in Figure 3. Cut-off values identified using Youden’s index were 3.5 for survival (64% sensitivity, 77.1% specificity, AUC 0.707) and 2.5 for disease relapse and local relapse (55.3% sensitivity, 79.9% specificity AUC 0.668; 65.5% sensitivity, 74.1% specificity, AUC 0.702, respectively). We can see that the values indicated by the ROC curves seem to be the same as the ones previously identified using survival analysis.

We split our patients into two groups for every cut-off and compared their overall, disease-free, and local relapse-free survival using Kaplan–Meier analysis. As shown in Figure 3, patients with less than 4 days of neoadjuvant treatment interruption have a 60-month survival probability of 90.2% (SE 2.1%), compared to 57.9% (SE 5.9%) for patients with 4 days or more of interruption. The group with less than 3 days of interruption fared better in terms of both DFS and LC: 82.2% (SE 2.8%) vs. 50.5% (SE 5.5%) and 94% (SE 1.9%) vs. 75.4% (SE 5.1%) respectively. All differences were statistically significant, with a *p*-value of <0.001.

Before computing a Cox proportional hazards model, we have to first check for predictor linearity, always true if the predictor is a binary categorical variable, and then test the proportional hazards assumption. For the latter, we computed Schoenfeld residuals using R. Only the model comprising overall survival and patients grouped around the 4-day mark satisfied both assumptions. The univariate Cox regression resulted in a hazard ratio of 5.89 (*p* < 0.001), showing that at any given moment, patients with 4 or more neoadjuvant interruption days are 5.89 times more likely to die than their counterparts with fewer gap days.

In order to further investigate the characteristics of the two groups we used a Mann–Whitney test to see if there is any difference in age distribution between people with an interruption of less than 4 days and 4 days or more, the result being statistically insignificant (*p* = 0.11). In terms of cTNM stage distribution, there is also no statistically significant difference between the groups (Chi-Square test *p*-value = 0.18). See additional Table A3.

### 3.3. Impact of Downstaging on Survival Curves

**Figure 4:**
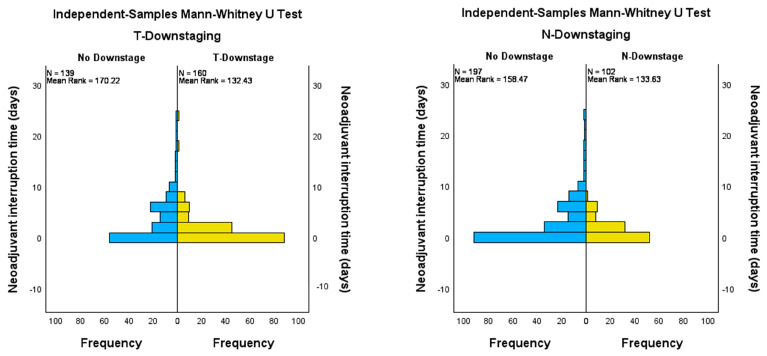
Mann–Whitney tests comparing the distribution of patients with no downstaging versus people with tumoral or nodal downstaging.

We compared the distribution of neoadjuvant therapy interruption days between patients with any type of downstage (nodal, tumoral) and no downstage. A Mann–Whitney test was thus used, showing statistical significance when comparing patients with tumoral downstaging and no tumoral downstage after neoadjuvant therapy (*p* < 0.001). A similar outcome was observed for nodal downstaging, with a *p*-value of 0.012 (Figure 4).

**Figure 5:**
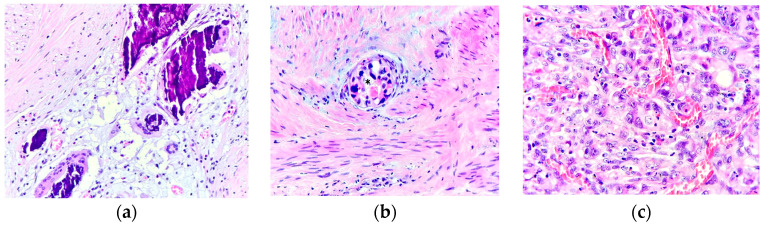
(**a**) HE × 400. Pathological complete response after neoadjuvant therapy, with tumor regression grade score 0 (Modified Ryan Scheme): no residual cancer cells: extensive fibrosis, microcalcifications, foamy histiocytes, and multinucleated giant cells. (**b**) HE × 400. Residual colorectal adenocarcinoma after neoadjuvant therapy with near complete response (tumor regression grade score 1, according to modified Ryan scheme): an isolated residual group of cancer cells (*), with a preponderance of fibrosis; (**c**) HE × 400. Residual colorectal adenocarcinoma-G3 after neoadjuvant therapy with no response (tumor regression grade score 3, according to Modified Ryan Scheme: extensive residual groups of cancer cells outgrowing fibrosis. HE-hematoxylin and eosin stain.

The mean number of interruption days was bigger for both patients without tumoral (3.52 days, SD 4.73 days) or nodal (3.06 days, SD 4.63) downstaging compared to patients that down-staged (1.47 days, SD 2.9 and 1.21 days, SD 1.7 respectively). Interruptions caused by COVID were usually long, ranging from 10 to 24 days, and none of the patients had tumoral or nodal downstaging. The difference between no response to neoadjuvant therapy (specimen from a patient with 12 interruption days due to COVID-19 infection), better response (specimen from a patient with 2 interruption days, showing tumoral downstaging), and complete response (specimen from a patient with no interruption) is illustrated in Figure 5.

**Figure 6:**
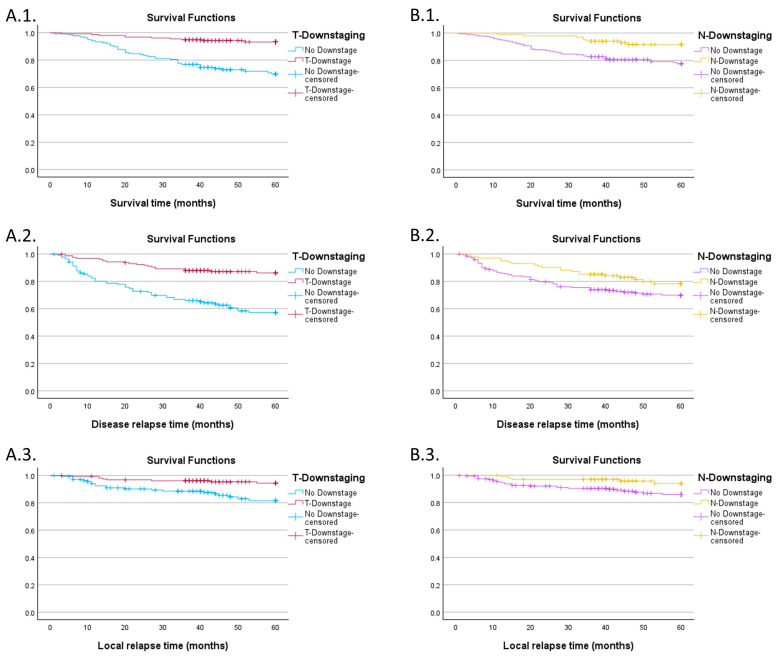
(**A.1**) Overall survival for patients grouped by tumoral downstaging; (**A.2**) DFS for patients grouped by tumoral downstaging; (**A.3**) LC for patients grouped by tumoral downstaging; (**B.1**) overall survival for patients grouped by nodal downstaging; (**B.2**) DFS for patients grouped by nodal downstaging; (**B.3**) LC for patients grouped by nodal downstaging.

In order to assess survival and relapse probability, we computed Kaplan–Meier curves for each outcome (overall survival, DFS, and time to local relapse), comparing the downstaging groups (Figure 6). Patients with no tumor downstaging had a lower 60-month survival probability regardless of the outcome evaluated (69.8% SE 4.1% for overall survival, 57.3% SE 4.5% for DFS and 81.7% SE 3.8% for local relapse) compared to patients that suffered tumoral downstaging (93.3% SE 2.1%, 86.3% SE 2.8% and 94.4% SE 2%, respectively), with significant log-rank tests (*p* < 0.001 for the first two, *p* = 0.001 for the third curve).

When grouping the population by nodal downstaging the only significant differences were observed for overall survival (77.6% SE 3.1% for the group without downstaging vs. 91.6% SE 2.9%) and LC (86% SE 2.7% vs. 94% SE 2.7%) with *p*-values of 0.003 and 0.037. For DFS, nodal downstaging seems to not be a statistically significant predictor (*p* = 0.063), with a probability of 70% (SE 3.4%) for patients with no downstaging compared to 78.3% (SE 4.4%) in the other group.

## 4. Discussion

Radiobiology is a field of basic research, specializing in understanding how high-energy ionizing radiation damages cancer cells and surrounding healthy tissue. In the case of rectal cancer, the general principles of radiobiology applicable to all neoplastic cells are followed, but with specific features for rectal adenocarcinoma, which has particular characteristics.

### 4.1. Radiobiology of Rectal Adenocarcinoma

Predicting biological effects after irradiation, a challenge since the discovery of X-rays, depends not only on the total dose but also on the specifics of treatment—fractional dose, dose rate, and duration of treatment. The linear-quadratic (LQ) model, validated by experimental and clinical data, is popular in radiotherapy for the management of clinical problems, such as adjustment of missed treatment, comparison of treatment regimens, and development of new schedules in clinical trials [24]. The α and β parameters in this model indicate the sensitivity of cells to radiation: higher values of these indicate increased sensitivity. The α/β ratio shows how sensitive cells are to the benefits of radiation fractionation: a higher ratio suggests reduced sensitivity to the benefits of fractionation. The LQ model is clinically essential to predict the benefits of fractionated radiotherapy and to compare the equivalent total doses of different fractionation schemes. The success of radiotherapy treatment and determination of the therapeutic window relies on accurate estimation of the LQ model parameters (α, β, and α/β ratio). Estimation of radiobiological parameters for neoplastic cells has been performed in hundreds of studies and a ratio α/β = 10 Gy is generally accepted. Most studies have focused on squamous cell carcinoma, tumor cells of nervous origin, and breast and prostate neoplasm. Relatively few radiobiological studies reveal the true α/β ratio of rectal adenocarcinoma. Suwinski et al. in a retrospective analysis of 168 patients showed an α value of 0.33 and β of 0.06 with an α/β ratio~5 Gy, well below the previously theorized value [25], thus a value sensitive to fractionation. In all studied schemes, the treatment duration was short, limiting the inclusion of the time effect in the model. The inclusion of time changed the α/β ratio to 11.1 Gy, a value without high fractionation sensitivity, and required a dose increase of 0.15 Gy/day to compensate for repopulation. These data explain why rectal neoplasm can be treated successfully and with similar results both hypofractionated and by conventional fractionation. The sensitivity of rectal cancer to radiation varies depending on several intrinsic and extrinsic factors, including the histological type of the tumor, the presence of certain genetic mutations, cell cycle characteristics, and microenvironmental conditions of the tumor. Understanding these factors allows physicians to better estimate tumor response to radiotherapy and to personalize treatment.

While radiation therapy is targeted against cancer cells, radiation can also affect healthy tissues around the tumor [26]. This can lead to a variety of side effects such as inflammation, fibrosis, and changes in bowel function. Side effects can vary depending on the radiation dose and the area treated, which is why doctors strive to balance the effective dose to destroy cancer cells while minimizing exposure to healthy tissues. Radiobiology research focuses not only on optimizing radiation therapy in the target but also on reducing these side effects. Side effects can vary depending on the radiation dose and the area treated, a problem partially solved by the introduction of IMRT technologies and the reduction of total irradiated volumes. A recent radiobiological study of rectal toxicity suggests that the rectum is not composed exclusively of serial functional subunits as theorized [27]. The study analyzed acute rectal toxicity data from 129 prostate cancer patients and observed that higher doses of radiation administered to rectal volumes of 15, 20, 30, and 40 cm^3^ are associated with a significant increase in acute rectal toxicity. In particular, higher doses for 30 and 40 cm^3^ volumes resulted in a 3.7 and 6-fold increase in the risk of acute toxicity, respectively. High radiation doses to small rectal volumes were not associated with significant increases in acute rectal toxicity, but irradiation of moderate rectal volumes with higher radiation doses led to a significant increase in the incidence of rectal toxicity with a steep dose gradient. This suggests the possibility that the rectum does not consist solely of parallel functional subunits, but of a combination of “mixed” or “serial-parallel” subunits. This is an important issue for the personalization of pelvic radiotherapy, as acute rectal symptoms are highly predictive of late toxicity [28]. In the case of late toxicity, a recent study recommends using a late rectal α/β ratio of no more than 3 Gy for prevention of proctitis, bleeding, and grade 1–2 sphincter control [29]. The same conclusions were drawn by Marzi et al. in a study published in 2009 [30] analyzing late rectal adverse reactions in prostate irradiated patients with stereotactic radiotherapy, results contradicting Brenner et al. who reported an α/β value of 5.4 with a margin of error of 1.5 Gy for late adverse reactions grade ≥2 [31].

### 4.2. Radiotherapy Dose Compensation

Personalized medicine in the treatment of rectal cancer is constantly evolving, offering treatment strategies based on individual tumor characteristics. This includes genetic and molecular analysis of tumors, in order to identify the most effective treatment approaches for each patient. Thus, tailoring radiation therapy is becoming a crucial factor in increasing success rates and reducing side effects [32]. Dose compensation lost in case of forced or unforced interruptions is a treatment personalization option that has been proposed and studied over the last decades to prevent inferior outcomes.

Since 1960, Elkind et al. have studied the effect of interruptions in ionizing radiation treatment and showed that extending the time of radiation administration leads to a lower tendency of cellular destruction due to sub-lethal damage repair [33]. Although the LQ model is used to calculate the effective biological dose and estimate cell survival, it does not take into account sub-lethal damage repaired due to extended dose delivery time. Kawahara et al. analyzed this phenomenon for discontinuation cases in photon radiotherapy and proposed a dose compensation method based on biological effectiveness [34]. They showed that sub-lethal damage repair has a significant contribution in decreasing the biological effectiveness of interrupted radiotherapy.

Putora et al. developed a simple method of calculating the dose needed to compensate for unfit interruptions in treatment. This online calculator is the basis of a compensability index defined by the authors, which takes into account both the desired tumor effect and the respect of normal tissue constraints [35]. Going even further, Abolfath et al. proposed a multi-scale mechanism to calculate the necessary corrections produced by interruptions taking into account both the DNA repair mechanism and the repopulation phenomenon [36].

A Russian study of patients with inoperable rectal malignancies who received combined radiation therapy (external gamma therapy and brachytherapy) had a significantly higher 4-year survival rate (40%) compared to those treated with external gamma therapy alone (18%) [37]. A lower incidence of acute reactions and late complications was also observed in the combination therapy group. The basis of these results lies in the LQ pattern and the total effective biological dose administered in the two groups (higher by approx. 15% in the brachytherapy group), suggesting that the addition of brachytherapy to the treatment regimen may significantly improve outcomes for patients with inoperable rectal cancer. Increasing the total effective biological dose by brachytherapy or compensating for the dose lost in external beam radiotherapy could be a new strategy to achieve pathological complete response (pCR) in rectal cancer. A 2017 review shows that although a superior pCR can be achieved by integrating brachytherapy, survival is not affected [38]. Fleischmann et al. however propose combining HDR brachytherapy with EBRT, especially in older, friable patients in whom a personalized strategy of omitting surgery may be an option in case of inoperability [39].

### 4.3. Impact of Radiotherapy Treatment Interruptions

One of the biggest problems with LCRT regimens is the risk of unplanned interruptions during the 25–28 treatment sessions, which is undesirable and can have a negative impact on the therapeutic goal. From a radiobiological point of view, the impact of interruptions can be calculated using mathematical methods based on the LQ model and the biological effective dose calculation [40]. Over the years, several radiobiological models have been studied to suggest the recovery of biological effective dose relative to total time and time lost, but these have limitations [41]. Clinical studies verifying the translation of these mathematical data into real life have been performed in several locations, but less so for rectal adenocarcinoma [42]. Repopulation is one of the basic elements of radiobiology and the 4Rs concept. This phenomenon represents the proliferation, often accelerated, of tumor cells that have survived after a dose of tumor-killing ionizing radiation. This repopulation is the main reason why treatment interruptions negatively affect LC and, indirectly, OS.

Despite these limitations, The Royal College of Radiologists has proposed some recommendations. If treatment has been interrupted without a doctor’s recommendation for a few days, the patient can receive therapy during the weekend so that there is no further deviation from the treatment plan. If this is not possible, two sessions per day can be held until the end of the treatment, to cover the missed dose due to the interruption of the treatment; if the double dose per day is possible, then it is recommended that the minimum time between the two sessions is 6 h. However, this decision should also be made according to the radiation dose the patient is receiving. Thus, if the radiotherapy fraction exceeds the value of 2.2 Gy, the approach of double therapy per day is no longer recommended. In case of unexpected discontinuation of therapy at a late stage of treatment, the compensation approach is modified. As mentioned above, models based on radiobiological calculations will be needed [43].

The radiobiology literature highlights the negative effects of suboptimal radiotherapy, regardless of its nature, which significantly impacts local control and patient survival. Nagar and Formenti report in their review that prolonging the time to adjuvant radiotherapy by more than 8 weeks after surgical approach in breast cancer has the effect of doubling the risk of local recurrence. Furthermore, data presented by the Early Breast Cancer Trialists’ Collaborative Group show that a reduction in the risk of local recurrence after radiotherapy correlates with a reduction in absolute risk concerning 15-year mortality [17]. However, Hanna et al. highlight in a recent systematic review the absence of studies with increased validity about the time from diagnosis to the start of neoadjuvant therapy in colorectal cancer or for several other curative radiotherapies [44,45]. Franssen et al. reinforce the lack of evidence from studies linking rectal cancer and delay of radiotherapy. They present in their systematic review the existence of 4 studies showing the relation between time to radiotherapy initiation and survival. However, only one study showed a decrease in survival if the prolongation from diagnosis to treatment initiation was more than 49 days [46]. In contrast, Bese et al. present in their review a study in which split-course radiotherapy was correlated with a decrease in locoregional control compared to the continuous course approach, even though the dose administered was higher [41]. Moreover, suboptimal and incomplete treatments may also impact the well-being of patients. The CROCODILE group study observed in their paper that patients who had poor adherence to treatment also had a decrease in monetary contribution from their own pockets in relation to treatment. Notably, however, this decrease, compared to patients who had complete therapy, was not associated with a decrease in the risk of catastrophic expenditure—defined as the total amount of capital out of pocket exceeding a certain percentage set by agreement [47].

The most studied neoplastic cell type from a radiobiological point of view is the squamous cell carcinoma of the head and neck, for which different treatment and compensation schemes have been proposed over the last 50 years. A split-course treatment scheme was proposed by a Danish trial published in 1988 which proposed a 3-week break in the middle of conventional treatment of 6.5 weeks, which resulted in a massive repopulation of clonogenic cells during this break and a decrease in survival from 41% to 30% in this group of patients [48]. Taking into account the aggressiveness of squamous cell carcinoma, accelerated radiotherapy regimens aiming at shortening the total treatment time were established and are still accepted today in certain head and neck cancer sites. A 5-day treatment break can decrease LC by up to 12% and a 10-day break by up to 20% according to a review published in 2007 [41,49,50]. Taking these findings further, it appears that the timing of interruptions throughout the radiotherapy cycle also plays a role in the negative effect: interruptions occurring in the first or last part of the treatment have a more pronounced impact than interruptions occurring in the middle of the treatment [51].

Similar to head and neck cancers, non-small cell carcinoma of the lung is negatively influenced by breaks in treatment. Each day of interruption can increase the chance of death by up to 2% [52]. Even concurrent chemotherapy does not improve OS in patients with a longer treatment interval [53].

Another localization was studied in 2001 and verified the benefit of the split course technique in anal carcinoma, concluding that a shorter break in the split technique was associated with a much lower recurrence rate, as much as 23.7 percent in younger patients [54]. Due to the poor results obtained for the split course regimen, the technique was abandoned altogether for anal cancer.

In cervical cancer, several studies have verified the relevance of prolonged treatment, because dual external-internal irradiation requires more organizational preparation and working time for medical staff: more simulations, treatment plans, dose calculations, and administrations. Most frequently, delays in treatment may occur after the first step (external radiotherapy) until brachytherapy is initiated. An increase in TTD from 6 weeks to 8 weeks correlates with a decrease in local control especially in stages IIB and III cervical cancer, with major concerns if brachytherapy is delayed more than one month after completion of external radiotherapy [55].

### 4.4. Reasons for Interruption

The responsibility for preventing potential interruptions in treatment lies with the radiation oncologist, who ought to plan as accurately as possible the radiotherapy sessions for rectal cancer patients and prevent any possible interruptions, especially those that can be predicted (such as holidays). Each of the reasons recorded in our study for interruptions will be considered in detail below.

1. Acute toxicities

The most common acute toxicities encountered in patients receiving rectal radio-chemotherapy appear in the epithelium, with the common manifestations being cystitis, dermatitis, or diarrhea. With technological advancement and the introduction of IMRT, adverse reactions due to local treatment have decreased significantly, with 2.7-fold decreases for IMRT being reported for diarrhea grade ≥ 2 and genitourinary toxicity grade ≥ 2 [56,57]. There are no major differences in acute toxicity between the conventional and hypofractionated radiotherapy regimens [58].

In the case of our study, 10% of patients experienced grade ≥ 2 acute toxicity, values that are close to those reported in a Japanese meta-analysis published in 2018 on a total of 859 patients [59]. However, we did not consider a sub-analysis of toxicities in our study due to the heterogeneity of the patient group, having performed both 2D, 3D-CRT, IMRT, and VMAT techniques, as well as 28.1% of patients not receiving chemotherapy.

Interruption of treatment due to acute toxicities negatively influences the overall duration of treatment, leading to changes in patient outcomes [60,61]. Acute adverse reactions can be discovered relatively quickly with careful follow-up of the patient by the radiation oncologist, which is why we recommend clinical consultation of the patient at least once a week during treatment. Prompt intervention with antidiarrheals, steroidal anti-inflammatories (for cystitis, proctitis), or topical treatments (for dermatitis) will prevent the need to interrupt radiotherapy. Also, preventing diarrhea by recommending a proper diet from the start of treatment can improve this goal. In the case of capecitabine chemotherapy, hematological monitoring is mandatory weekly, and any hematological deterioration can be compensated for by stopping neoadjuvant chemotherapy only, without stopping radiotherapy, according to the clinic protocol.

2. The impact of the COVID-19 pandemic

The beginning of December 2019 brought a new global challenge, due to the infection of the coronavirus called SARS-CoV2. In addition to the devastating effects, it has had directly through respiratory infection, the pandemic has indirectly caused hard-to-estimate damage to the cancer population through massive delays in treatment. The national protocol in Romania initially provided for a 14-calendar day isolation for infected patients. After the introduction of the vaccine, this isolation period decreased first to 10 days, then to 5 days for the vaccinated population. At the same time, patients were ensured uninterrupted access to oncological treatments, except for a 2-month period in March–May 2020 when surgeries were severely affected. During this period, LARC patients who became infected during neoadjuvant radio-chemotherapy were compulsorily isolated and treatment was interrupted.

In our group, 9 patients interrupted treatment for at least 10 days due to SARS-CoV-2 infection. This long interruption should normally be compensated for by dose supplementation, but the presence of comorbidities due to respiratory infection barely allowed the completion of the initially prescribed dose. In other centers in Europe, radiotherapy was not necessarily interrupted by the presence of infection, but epidemiological circuits were organized for infected patients so that all those with mild forms of the disease were able to carry out treatment sessions at the end of the workday [62]. This was likely beneficial from an oncological point of view for the patient, at the expense of a higher risk for exposed medical staff or even for other patients already infected through exposure to other strains of the virus.

3. Machine failure

In our study, a common reason for interruptions is LINAC failure. Most patients received radiotherapy in a public hospital in Romania. Equipment problems can be put both on the overuse of the machines, a problem that was very common until 2010–2020, but also on the poor maintenance. These problems have been partly solved in recent years, with the opening of more private centers and the improvement of radiotherapy funding. Romania is considered a developed country starting from 2019, with a drop back to the middle class in 2020 post-pandemic and a recovery from 2021 that seems stationary. Several comparative studies have shown that countries in the low- and middle-income categories are affected in terms of consistent and predictable funding in the health system, which has a direct impact on keeping radiotherapy machines in optimal parameters. In Indonesia, a downtime of radiotherapy machines of up to 23% per year has been observed in some centers [63,64], well above the 1–2% per year promised by linear accelerator manufacturers. In Romania, at the time of publication of this study, no official statistics show the degree of downtime caused by the failure of radiotherapy equipment.

4. Days off

No less than 42 patients in our study discontinued neoadjuvant treatment because of public holidays. Romania has 15 days off per year, to which extra days off are frequently added, in order to create a longer holiday when a public holiday falls on a Tuesday or a Thursday. In these situations, the public radiotherapy clinics have to improvise so as not to interrupt treatments. The only effective strategy is to anticipate these days off and to plan the schedules so that treatment does not stop during holidays. Another potential strategy for some patients is to perform hypofractionation, an SCRT schedule lasting only one week. Another option is for radiotherapy units to voluntarily exchange days off if they fall in the middle of the week with one day at the end of the week to ensure continuity of treatment. Other countries have a significantly higher number of days off, such as Egypt with 22 statutory days off, or Nepal with 35 days [65], which have even greater challenges to ensure continuity of cancer treatment.

5. Other reasons

In the context of interruption of cancer treatment, multiple causes can be found. Discontinuation of oncological intervention affects not only the evolution of the patient’s disease but also the quality of studies, especially prospective clinical trials, which help in the development of optimal treatment measures. Sidani et al. report that patient preference for a particular type of therapy influences both treatment adherence and outcome following treatment. Thus, low adherence may negatively influence the validity of intervention studies [66]. This is also supported by Leykin et al. They show that the patient can be affected in two ways following randomization. Firstly, the patient is limited in decision-making, which may lead to increased demoralization and decreased curative capacity of the body. Secondly, patient perception of the efficacy of a treatment may increase commitment to therapy, and concomitantly improve outcome [67]. In addition, Diefenhardt et al. highlight the lack of studies correlating treatment adherence and oncological outcome in phase 3 clinical trials in rectal cancer. Their study, an extension to the CAO/ARO/AIO-04 trial, shows a significant association between complete adherence and poor adherence to neoadjuvant therapy. In addition, the study also presents the importance of optimal dosing and implementation of supportive therapies to improve adherence to neoadjuvant therapy in rectal cancer, especially in elderly patients and those with poor performance status [68]. Compared to the data presented above, Jung et al. observed in their review a positive performance of TNT strategies. Thus, patients showed better adherence to the “consolidation TNT protocol” when compared to radiotherapy (97% vs. 92%) [69].

Symptoms caused by increased toxicity may also result in decreased adherence to treatment [70]. Gebert et al. report in their study that the rate of treatment discontinuation due to patient choice is between 30% and 50%. Low values on quality of life (QoL) scales result in an increased risk of dropping out of cancer treatment. Thus, decreased scores reported on the subscales “role functioning”, “physical functioning”, and “fatigue symptom”, assessed by the EORTC-C30 questionnaire were key elements that caused the patient to discontinue cancer treatment. In addition, low social support was associated with a 2-fold increased risk for treatment discontinuation [71,72]. Furthermore, Stalmeier et al. expose that in clinical practice, the treating physician tends to take ownership of therapeutic decisions, so that the patient does not question the physician about oncological decisions. Thus, the doctor was not able to determine which patient would like to choose their own treatment, but neither was the patient able to predict which treatment would be given. For example, 71% of patients preferred the low-toxicity regimen, whereas only 51% of treating physicians made the same choice [73].

Patient compliance and personalized assessment of acute toxicities contribute to achieving the goal of uninterrupted treatment [74]. The role of psychological and nutritional support teams that can predict and manage potentially disruptive problems early on is all the more important in the era of intensified non-surgical cancer treatments.

### 4.5. The Role of Total Neoadjuvant Treatment

Several TNT regimens are currently being studied, the largest groups being in the RAPIDO study with almost 1000 patients, STELLAR with 591 patients, and POLISH II with 515 patients. All these schemes use short-course hypofractionated radiotherapy with a total dose of 25 Gy in 5 fractions as the first treatment in sequence, followed by mandatory chemotherapy with oxaliplatin and an antimetabolite (5-FU or Capecitabine), then surgery [75,76,77]. The PRODIGE-23 trial involved LCRT and the addition of irinotecan to FOLFOX [78,79]. All these studies have shown good 3-year survivals between 87–91% and local or distant 3-year recurrences of 65–76%, except POLISH II, which included only patients with advanced tumors [15]. A significantly higher pCR was observed, approximately double, that in the standard neoadjuvant treatment used as a comparator in all these studies. The OPRA study, however, addressed a new tactic of omitting radical surgery in patients with pCR [8]. This “watch and wait” strategy involves a much closer follow-up of the patient during and after treatment. Therefore, postponing surgery (in those for whom local relapse eventually occurs) or complete omission has a positive impact on the patient’s QoL and perception of cancer care. A final category investigated was the order of neoadjuvant chemotherapy versus neoadjuvant radiotherapy, with results partially in favor of consolidation chemotherapy (and thus starting the treatment order with radiotherapy), which was studied in particular in the German CAO/ARO/AIO-12 [80,81] and OPRA [9] trials.

A major advantage that the authors of this study see for the potential of TNT strategies to modify future rectal cancer guidelines (as presented by Nurkin at the National Comprehensive Cancer Network 2023 Annual Conference) is the change in radiotherapy regimens from conventional to hypofractionated regimens. This approach may have advantages in terms of unburdening radiotherapy units, a concern that is even more pressing in developing countries [63], and in terms of reducing the number of days of treatment interruption. A 5-day treatment—carried out from Monday to Friday—is more likely to be completed without interruptions through proper planning by the radiation oncologist to avoid public holidays or potential scheduled maintenance interventions to the LINAC. Also, the shortening from 5–6 weeks to just 1 week of treatment will see better adherence in patients who do not have to come to the hospital as often [82], as well as in the chance of having fewer acute side effects during treatment. Although a meta-analysis published in 2021 [58] shows that acute toxicity differences are not significant between the LCRT and SCRT regimen, adverse reactions of rectal cancer radiotherapy generally occur after 1–2 weeks [83], which would mean that patients undergoing SCRT are more likely not to interrupt treatment due to acute toxicity. Increased adherence to radiotherapy in the TNT regimen was noted in a recent article highlighting the advantages of TNT regimens using SCRT [69].

### 4.6. Study Limitations

Firstly, this is a retrospective study that includes only patients who had a total dose of neoadjuvant radiotherapy in the range of 45–50.4 Gray. It should be taken into account that a large proportion of patients who had many days of interruption also received dose compensation as recommended by experts. We selected in this study only those patients who did not exceed the total dose of 50.4 Gray, which is why only 299 patients met the inclusion criteria after the analysis of the registers. We chose to go back in the registries until 2004, in order to have a large enough group of patients with discontinuations to obtain meaningful results, which brought as a disadvantage the use of 2D radiotherapy techniques or the inclusion of some patients who did not receive neoadjuvant chemotherapy, thus making the group of patients more heterogeneous.

Secondly, the selection of reasons for interruption was based on the most commonly reported comments in the medical documents. In some cases, this was expressed as “patient refusal”, which was included in the “other” category that takes into account both patient non-compliance and their personal reasons or other undefined causes. This subcategory of reasons is also the most frequently encountered.

Lastly, we chose to select patients up to and including 2020 to cover the unfavorable period during the COVID-19 pandemic. These patients had not yet reached the follow-up target of 60 months and were censored at the time of the study if the defining event was not reached. The authors aim to continue this study by evaluating the negative impact of the COVID-19 pandemic on cancer patients who had difficult access to cancer treatments during those years.

## 5. Conclusions

Interruptions in neoadjuvant treatment have a negative impact on probabilities for OS, LC, and DFS. In the case of OS, we identified that patients who have 4 or more days of discontinuation are most affected, while for LC and DFS, the statistical cut-off point is 3 days.

The appearance of tumor and nodal downstaging is inversely proportional to the number of days of discontinuation. Patients with tumor downstaging had a higher probability of OS, LC, and DFS than those without downstaging, whereas those with nodal downstaging scored better only on OS and LC.

The authors strongly recommend that radiation oncologists identify and anticipate potential causes of treatment interruptions, both unforced ones such as holidays, maintenance, or device failure, and forced ones such as toxicities or poor treatment adherence. Preventing these interruptions will maximize the chances of achieving optimal outcomes for our patients.

## Figures and Tables

**Table 1:** General characteristics of our population.

Variable	Number			Frequency (%)
Sex				
Male	172			57.53
Female	127			42.47
cTNM				
II	101			33.78
III	198			66.22
ypTNM				
0	30			10.03
I	84			28.10
II	70			23.41
III	115			38.46
Neoadjuvant therapy				
RT	84			28.09
RT + CHT	215			71.90
Tumor location				
Low rectum	142			47.49
Mid rectum	111			37.12
Upper rectum	46			15.39
Radiotherapy technique				
2D	103			34.45
3D	53			17.73
IMRT	143			47.82
Radiation dose				
4500 cGrays	167			55.85
5000 cGrays	41			13.71
5040 cGrays	91			30.44
Tumor downstaging				
Yes	139			46.49
No	160			53.51
Nodal downstaging				
Yes	197			65.89
No	102			34.11
Neoadjuvant interruption				
0 days	144			48.16
1 day	37			12.38
2 days	29			9.70
3 days	16			5.35
4 days	7			2.34
5 days	25			8.36
6+ days	41			13.71
Reason for interruption		Mean	SD	
Holiday	42	2 days	1.3	26.92
Toxicity	30	5.1 days	1.9	19.23
Machine failure	18	2.4 days	1.5	11.54
COVID	9	15.9 days	4.8	5.77
Other reasons	57	5.3 days	4.6	36.54

## Data Availability

Data are available only upon request due to ethical restrictions. The data presented in this study are available upon request from the main or corresponding author and the Colțea Clinical Hospital (secretariat@coltea.ro). The data are not publicly available due to the policy of Colțea Clinical Hospital to have the approval of the Ethics Committee for each new research study.

## Appendix A

**Table A1:** Overall survival, disease-free survival, and local control probabilities by ypTNM stage.

ypTNM Stage	Overall Survival		Disease-Free Survival		Local Control	
	Estimate	SE	Estimate	SE	Estimate	SE
0	95%	4.9%	91%	6.3%	94.1%	5.7%
I	98.3%	1.7%	96.4%	2%	98.8%	1.2%
II	86.9%	4.5%	65.3%	6.4%	83.2%	5.2%
III	64.5%	4.5%	54.3%	4.8%	82.8%	3.7%

SE—standard error.

**Table A2:** Overall survival, disease-free survival, and local control probabilities by days of neoadjuvant therapy interruption.

Days of Interruption	Overall Survival		Disease-Free Survival		Local Control	
	Estimate	SE	Estimate	SE	Estimate	SE
0 days	88.8%	2.9%	80.5%	3.6%	93%	2.4%
1 day	91.2%	4.9%	94.3%	3.9%	na	na
2 days	96.6%	3.4%	75.5%	8.1%	92.5%	5.1%
3 days	87.5%	8.3%	52.1%	13.9%	67%	14.9%
4 days	71.4%	17.1%	57.1%	18.7%	na	na
5 days	50.3%	10.3%	43.6%	10%	81.4%	8.7%
6+ days	61%	7.6%	53.2%	8.1%	69.9%	7.7%

SE—Standard Error.

**Table A3:** Contingency table showing the cTNM stage distribution for people with less than 4 days vs. 4 days or more of neoadjuvant therapy interruptions.

	cTNM		Total
	II	III	
Under 4 days	81	145	226
4 days or more	20	53	73
Total	101	198	299

## References

[1] Rahbari N.N., Elbers H., Askoxylakis V., Motschall E., Bork U., Büchler M.W., Weitz J., Koch M. (2013). Neoadjuvant radiotherapy for rectal cancer: Meta-analysis of randomized controlled trials. Ann. Surg. Oncol..

[2] Hashiguchi Y., Muro K., Saito Y., Ito Y., Ajioka Y., Hamaguchi T., Hasegawa K., Hotta K., Ishida H., Ishiguro M. (2020). Japanese Society for Cancer of the Colon and Rectum (JSCCR) guidelines 2019 for the treatment of colorectal cancer. Int. J. Clin. Oncol..

[3] Benson A.B., Venook A.P., Al-Hawary M.M., Azad N., Chen Y.J., Ciombor K.K., Cohen S., Cooper H.S., Deming D., Farkas L. (2023). NCCN Guidelines Version 4.2022 Rectal Cancer Continue NCCN Guidelines Panel Disclosures. https://www.nccn.org/professionals/physician_gls/pdf/rectal.pdf.

[4] Glynne-Jones R., Wyrwicz L., Tiret E., Brown G., Rödel C., Cervantes A., Arnold D. (2017). Rectal cancer: ESMO Clinical Practice Guidelines for diagnosis, treatment and follow-up. Ann. Oncol..

[5] Liscu H.-D., Liscu B.-R., Mitre R., Anghel I.-V., Antone-Iordache I.-L., Balan A., Coniac S., Miron A.-I., Halcu G. (2023). The Conditioning of Adjuvant Chemotherapy for Stage II and III Rectal Cancer Determined by Postoperative Pathological Characteristics in Romania. Medicina.

[6] Enker W.E. (1997). Total mesorectal excision—The new golden standard of surgery for rectal cancer. Ann. Med..

[7] Heald R.J., Ryall R.D.H. (1986). Recurrence and Survival after Total Mesorectal Excision for Rectal Cancer. Lancet.

[8] Garcia-Aguilar J., Patil S., Kim J.K., Yuval J.B., Thompson H., Verheij F., Lee M., Saltz L.B. (2020). Preliminary results of the organ preservation of rectal adenocarcinoma (OPRA) trial. J. Clin. Oncol..

[9] Garcia-Aguilar J., Patil S., Gollub M.J., Kim J.K., Yuval J.B., Thompson H.M., Verheij F.S., Omer D.M., Lee M., Dunne R.F. (2022). Organ Preservation in Patients with Rectal Adenocarcinoma Treated with Total Neoadjuvant Therapy. J. Clin. Oncol..

[10] Jimenez-Rodriguez R.M., Quezada-Diaz F., Hameed I., Kalabin A., Patil S., Smith J.J., Garcia-Aguilar J.M. (2021). Organ Preservation in Patients with Rectal Cancer Treated with Total Neoadjuvant Therapy. Dis. Colon Rectum.

[11] Bach S.P., Gilbert A., Brock K., Korsgen S., Geh I., Hill J., Gill T., Hainsworth P., Tutton M.G., Khan J. (2021). Radical surgery versus organ preservation via short-course radiotherapy followed by transanal endoscopic microsurgery for early-stage rectal cancer (TREC): A randomised, open-label feasibility study. Lancet Gastroenterol. Hepatol..

[12] Liang J.-A., Kuo Y.-C., Chao K.C., Chen W.T.-L., Ke T.-W., Chou S.-H., Li C.-C., Chien C.-R. (2022). High vs. Standard Radiotherapy Dose in Locally Advanced Rectal Adenocarcinoma Patients Treated with Neoadjuvant Long Course Chemoradiotherapy: A Population-based Study. Anticancer Res..

[13] McCarthy K., Pearson K., Fulton R., Hewitt J. (2012). Pre-operative chemoradiation for non-metastatic locally advanced rectal cancer. Cochrane Database Syst. Rev..

[14] Glynne-Jones R., Hollingshead J. (2023). TNT and local recurrence in the RAPIDO trial—Untangling the puzzle. Nat. Rev. Clin. Oncol..

[15] Johnson G.G., Park J., Helewa R.M., Goldenberg B.A., Nashed M., Hyun E. (2023). Total neoadjuvant therapy for rectal cancer: A guide for surgeons. Can. J. Surg..

[16] Liu S., Jiang T., Xiao L., Yang S., Liu Q., Gao Y., Chen G., Xiao W. (2021). Total Neoadjuvant Therapy (TNT) versus Standard Neoadjuvant Chemoradiotherapy for Locally Advanced Rectal Cancer: A Systematic Review and Meta-Analysis. Oncologist.

[17] Nagar H., Formenti S.C. (2020). Cancer and COVID-19—Potentially deleterious effects of delaying radiotherapy. Nat. Rev. Clin. Oncol..

[18] Ciarleglio F.A., Rigoni M., Mereu L., Tommaso C., Carrara A., Malossini G., Tateo S., Tirone G., Johansen T.E.B., Benetollo P.P. (2021). The negative effects of COVID-19 and national lockdown on emergency surgery morbidity due to delayed access. World J. Emerg. Surg..

[19] DeMore N.K., Savage S.J., Lesher A.P., Abbott A., Giordano A., Zhang B., Mahvi D.M., Garcia D., Carneiro-Pla D., Camp E.R. (2020). What is Elective Oncologic Surgery in the Time of COVID-19? A Literature Review of the Impact of Surgical Delays on Outcomes in Patients with Cancer. Clin. Oncol. Res..

[20] RHunger R., König V., Stillger R., Mantke R. (2022). Impact of the COVID-19 pandemic on delays in surgical procedures in Germany: A multi-center analysis of an administrative registry of 176,783 patients. Patient Saf. Surg..

[21] Treder M., Vogelsang R.P., Janssen S., Schild S.E., Holländer N.H., Rades D. (2018). Potential Prognostic Factors of Downstaging Following Preoperative Chemoradiation for High Rectal Cancer. In Vivo.

[22] Chen S., Tang Y., Li N., Liu W., Jiang J., Jiang L., Chen B., Fang H., Ren H., Lu N. (2020). Neoadjuvant Rectal Score And Downstaging Depth Score as Prognosis Predictors For Patients with Locally Advanced Rectal Cancer. Int. J. Radiat. Oncol. Biol. Phys..

[23] Valentini V., Coco C., Picciocchi A., Morganti A.G., Trodella L., Ciabattoni A., Cellini F., Barbaro B., Cogliandolo S., Nuzzo G. (2002). Does downstaging predict improved outcome after preoperative chemoradiation for extraperitoneal locally advanced rectal cancer? A long-term analysis of 165 patients. Int. J. Radiat. Oncol. Biol. Phys..

[24] Sardari D., Verga N. (2010). Calculation of externally applied electric field intensity for disruption of cancer cell proliferation. Electromagn. Biol. Med..

[25] Suwinski R., Wzietek I., Tarnawski R., Namysl-Kaletka A., Kryj M., Chmielarz A., Wydmanski J. (2007). Moderately Low Alpha/Beta Ratio for Rectal Cancer May Best Explain the Outcome of Three Fractionation Schedules of Preoperative Radiotherapy. Int. J. Radiat. Oncol. Biol. Phys..

[26] Groza A., Iconaru S.L., Jiga G., Chapon P., Gaiaschi S., Verga N., Beuran M., Prodan A.M., Matei M., Marinescu S.A. (2019). The Effect of the Ionizing Radiation on Hydroxyapatite–Polydimethylsiloxane Layers. Polym. Eng. Sci..

[27] Masters B., Pascoe A., Walker G., Sundar S. (2018). Radiobiology of Acute Rectal Toxicity. Clin. Oncol..

[28] Lemanska A., Dearnaley D., Jena R., Sydes M., Faithfull S. (2018). Older Age, Early Symptoms and Physical Function are Associated with the Severity of Late Symptom Clusters for Men Undergoing Radiotherapy for Prostate Cancer. Clin. Oncol..

[29] Brand D.H., Brüningk S.C., Wilkins A., Fernandez K., Naismith O., Gao A., Syndikus I., Dearnaley D.P., Tree A.C., van As N. (2021). Estimates of Alpha/Beta (α/β) Ratios for Individual Late Rectal Toxicity Endpoints: An Analysis of the CHHiP Trial. Int. J. Radiat. Oncol. Biol. Phys..

[30] Marzi S., Saracino B., Petrongari M.G., Arcangeli S., Gomellini S., Arcangeli G., Benassi M., Landoni V. (2009). Modeling of αβ for late rectal toxicity from a randomized phase II study: Conventional versus hypofractionated scheme for localized prostate cancer. J. Exp. Clin. Cancer Res..

[31] Brenner D.J. (2004). Fractionation and late rectal toxicity. Int. J. Radiat. Oncol. Biol. Phys..

[32] Georgescu M.-I., Ionescu R.T., Miron A.-I., Savencu O., Ristea N.-C., Verga N., Khan F.S. Multimodal Multi-Head Convolutional Attention with Various Kernel Sizes for Medical Image Super-Resolution. Proceedings of the 2023 IEEE Winter Conference on Applications of Computer Vision, WACV 2023.

[33] Elkind M.M., Sutton H. (1960). Radiation response of mammalian cells grown in culture. 1. Repair of X-ray damage in surviving Chinese hamster cells. Radiat. Res..

[34] Kawahara D., Nakano H., Saito A., Ozawa S., Nagata Y. (2020). Dose compensation based on biological effectiveness due to interruption time for photon radiation therapy. Br. J. Radiol..

[35] Putora P.M., Schmuecking M., Aebersold D., Plasswilm L. (2012). Compensability index for compensation radiotherapy after treatment interruptions. Radiat. Oncol..

[36] Abolfath R., Khalili M., Senejani A.G., Kodery B., Ivker R. (2022). The Dependence of Compensation Dose on Systematic and Random Interruption Treatment Time in Radiation Therapy. Onco.

[37] Atkočius V., Burneckis A., Atkočiené E. (1995). Clinical radiobiology of HDR Cf-252 brachytherapy for rectal cancer. Radiother. Oncol..

[38] Buckley H., Wilson C., Ajithkumar T. (2017). High-Dose-Rate Brachytherapy in the Management of Operable Rectal Cancer: A Systematic Review. Int. J. Radiat. Oncol. Biol. Phys..

[39] Fleischmann M., Diefenhardt M., Trommel M., Scherf C., Ramm U., Chatzikonstantinou G., Fokas E., Rödel C., Tselis N. (2022). Image-guided high-dose-rate brachytherapy for rectal cancer: Technical note and first clinical experience on an organ-preserving approach. Strahlenther. Onkol..

[40] Sinclair J.A., Oates J.P., Dale R.G. (1999). BED-time charts and their application to the problems of interruptions in external beam radiotherapy treatments. Int. J. Radiat. Oncol. Biol. Phys..

[41] Bese N.S., Hendry J., Jeremic B. (2007). Effects of Prolongation of Overall Treatment Time Due To Unplanned Interruptions During Radiotherapy of Different Tumor Sites and Practical Methods for Compensation. Int. J. Radiat. Oncol. Biol. Phys..

[42] Giuglea C., Marin A., Gavrila I., Paunescu A., Dobrete N.A., Marinescu S.A. (2023). Basal Cell Carcinoma—A Retrospective Descriptive Study Integrated in Current Literature. Life.

[43] The Royal College of Radiologists (2019). The Timely Delivery of Radical Radiotherapy: Guidelines for the Management of Unscheduled Treatment Interruptions.

[44] Hanna T.P., King W.D., Thibodeau S., Jalink M., Paulin G.A., Harvey-Jones E., O’Sullivan D.E., Booth C.M., Sullivan R., Aggarwal A. (2020). Mortality due to cancer treatment delay: Systematic review and meta-analysis. BMJ.

[45] Popescu-Vâlceanu H.-C., Stoicea M.C., Enache V., Bratu R.M., Mustăţea P., Drăguţ R.M., Rusu E., Ionescu-Tîrgovişte C., Radulian G. (2022). Bcl-2 and p53 immunophenotypes in colorectal adenocarcinoma in type 2 diabetes mellitus versus non-diabetic patients. Rom. J. Morphol. Embryol..

[46] Franssen R.F.W., Strous M.T.A., Bongers B.C., Vogelaar F.J., Janssen-Heijnen M.L.G. (2021). The Association Between Treatment Interval and Survival in Patients with Colon or Rectal Cancer: A Systematic Review. World J. Surg..

[47] CROCODILE Study Group (2022). Catastrophic expenditure and treatment attrition in patients seeking comprehensive colorectal cancer treatment in India: A prospective multicentre study. Lancet Reg. Health-Southeast Asia.

[48] Overgaard J., Hjelm-Hansen M., Johansen L.V., Andersen A.P. (1988). Comparison of conventional and split-course radiotherapy as primary treatment in carcinoma of the larynx. Acta Oncol..

[49] Barton M.B., Keane T.J., Gadalla T., Maki E. (1992). The effect of treatment time and treatment interruption on tumour control following radical radiotherapy of laryngeal cancer. Radiother. Oncol..

[50] Maciejewski B., Preuss-Bayer G., Trott K.-R. (1983). The influence of the number of fractions and of overall treatment time on local control and late complication rate in squamous cell carcinoma of the larynx. Int. J. Radiat. Oncol. Biol. Phys..

[51] Skladowski K., Law M.G., Maciejewski B., Steel G.G. (1994). Planned and unplanned gaps in radiotherapy: The importance of gap position and gap duration. Radiother. Oncol..

[52] Cox J.D., Pajak T.F., Asbell S., Russell A.H., Pederson J., Byhardt R.W., Emami B., Roach M. (1993). Interruptions of high-dose radiation therapy decrease long-term survival of favorable patients with unresectable non-small cell carcinoma of the lung: Analysis of 1244 cases from 3 Radiation Therapy Oncology Group (RTOG) trials. Int. J. Radiat. Oncol. Biol. Phys..

[53] Machtay M., Hsu C., Komaki R., Sause W.T., Swann R.S., Langer C.J., Byhardt R.W., Curran W.J. (2005). Effect of overall treatment time on outcomes after concurrent chemoradiation for locally advanced non-small-cell lung carcinoma: Analysis of the Radiation Therapy Oncology Group (RTOG) experience. Int. J. Radiat. Oncol. Biol. Phys..

[54] Weber D.C., Kurtz J.M., Allal A.S. (2001). The impact of gap duration on local control in anal canal carcinoma treated by split-course radiotherapy and concomitant chemotherapy. Int. J. Radiat. Oncol. Biol. Phys..

[55] Lanciano R.M., Pajak T.F., Martz K., Hanks G.E. (1993). The influence of treatment time on outcome for squamous cell cancer of the uterine cervix treated with radiation: A patterns-of-care study. Int. J. Radiat. Oncol. Biol. Phys..

[56] Samuelian J.M., Callister M.D., Ashman J.B., Young-Fadok T.M., Borad M.J., Gunderson L.L. (2012). Reduced Acute Bowel Toxicity in Patients Treated with Intensity-Modulated Radiotherapy for Rectal Cancer. Int. J. Radiat. Oncol. Biol. Phys..

[57] Ng S.Y., Colborn K.L., Cambridge L., Hajj C., Yang T.J., Wu A.J., Goodman K.A. (2016). Acute toxicity with intensity modulated radiotherapy versus 3-dimensional conformal radiotherapy during preoperative chemoradiation for locally advanced rectal cancer. Radiother. Oncol..

[58] Liscu H.-D., Miron A.-I., Rusea A.-R., Oprea A.-M.N., Mitre R., Herdea A., Negreanu R. (2021). Short-Course Radiotherapy versus Long-Course Radio-Chemotherapy as Neoadjuvant Treatment for Locally Advanced Rectal Cancer: Meta-Analysis from a Toxicity Perspective. Maedica.

[59] Wee C.W., Kang H.-C., Wu H.-G., Chie E.K., Choi N., Park J.M., Kim J.-I., Huang C.-M., Wang J.-Y., Ng S.Y. (2018). Intensity-modulated radiotherapy versus three-dimensional conformal radiotherapy in rectal cancer treated with neoadjuvant concurrent chemoradiation: A meta-analysis and pooled-analysis of acute toxicity. Jpn. J. Clin. Oncol..

[60] Coniac S., Outas M.C.C., Pirvu E.-E., Patru R.-I., Gainariu E., Aldea C., Iorga P.G., Ambroci M., Liscu H.-D., Miron A.-I. (2023). Challenges and Limitations of Endocrine Toxicity Evaluation in Non-Small Cell Lung Cancer Patients Treated with Immunotherapy-Retrospective Study from a Tertiary-Level Hospital in Romania. Diagnostics.

[61] Grigorean V.T., Erchid A., Coman I.S., Liţescu M. (2023). Colorectal Cancer—The ‘Parent’ of Low Bowel Obstruction. Medicina.

[62] Bernabucci L., Cornacchione P., Boldrini L., Pasini D., Dinapoli L., Smiljanic L., Valentini V., Dinapoli N. (2022). Radiotherapy during the COVID-19: A review about management and treatment strategies. Rep. Pract. Oncol. Radiother..

[63] Peiris G.S., Pawiro S.A., Kasim M.F., Sheehy S.L. (2023). Failure modes and downtime of radiotherapy LINACs and multileaf collimators in Indonesia. J. Appl. Clin. Med. Phys..

[64] Wroe L., Ige T., Asogwa O., Aruah S., Grover S., Makufa R., Fitz-Gibbon M., Sheehy S. (2020). Comparative Analysis of Radiotherapy Linear Accelerator Downtime and Failure Modes in the UK, Nigeria and Botswana. Clin. Oncol..

[65] Countries with the Most Public Holidays. WorldAtlas. https://www.worldatlas.com/articles/countries-with-the-most-public-holidays.html.

[66] Sidani S., Fox M., Streiner D.L., Miranda J., Fredericks S., Epstein D.R. (2015). Examining the influence of treatment preferences on attrition, adherence and outcomes: A protocol for a two-stage partially randomized trial. BMC Nurs..

[67] Leykin Y., DeRubeis R.J., Gallop R., Amsterdam J.D., Shelton R.C., Hollon S.D. (2007). The relation of patients’ treatment preferences to outcome in a randomized clinical trial. Behav. Ther..

[68] Diefenhardt M., Ludmir E.B., Hofheinz R.-D., Ghadimi M., Minsky B.D., Rödel C., Fokas E. (2020). Association of Treatment Adherence with Oncologic Outcomes for Patients with Rectal Cancer: A Post Hoc Analysis of the CAO/ARO/AIO-04 Phase 3 Randomized Clinical Trial. JAMA Oncol..

[69] Jung K.U., Kim H.O., Kim H., Lee D., Cheong C. (2023). Unveiling the profound advantages of total neoadjuvant therapy in rectal cancer: A trailblazing exploration. Ann. Surg. Treat. Res..

[70] Ionescu A.-I., Atasiei D.-I., Ionescu R.-T., Ultimescu F., Barnonschi A.-A., Anghel A.-V., Anghel C.-A., Antone-Iordache I.-L., Mitre R., Bobolocu A.M. (2024). Prediction of Subclinical and Clinical Multiple Organ Failure Dysfunction in Breast Cancer Patients-A Review Using AI Tools. Cancers.

[71] Gebert P., Schindel D., Frick J., Schenk L., Grittner U. (2021). Characteristics and patient-reported outcomes associated with dropout in severely affected oncological patients: An exploratory study. BMC Med. Res. Methodol..

[72] Mehedintu C., Frincu F., Brinduse L.A., Carp-Veliscu A., Bratila E., Hennetier C., Roman H. (2021). Postoperative Assessment of the Quality of Life in Patients with Colorectal Endometriosis. J. Clin. Med..

[73] Stalmeier P.F., van Tol-Geerdink J.J., van Lin E.N., Schimmel E., Huizenga H., van Daal W.A., Leer J.-W. (2007). Doctors’ and patients’ preferences for participation and treatment in curative prostate cancer radiotherapy. J. Clin. Oncol..

[74] Miron A.-I., Anghel A.-V., Barnonschi A.-A., Mitre R., Liscu H.-D., Găinariu E., Pătru R., Coniac S. (2023). Real-World Outcomes of CDK4/6 Inhibitors Treatment in Metastatic Breast Cancer in Romania. Diagnostics.

[75] Bahadoer R.R., Dijkstra E.A., van Etten B., Marijnen C.A.M., Putter H., Kranenbarg E.M.-K., Roodvoets A.G.H., Nagtegaal I.D., Beets-Tan R.G.H., Blomqvist L.K. (2020). Short-course radiotherapy followed by chemotherapy before total mesorectal excision (TME) versus preoperative chemoradiotherapy, TME, and optional adjuvant chemotherapy in locally advanced rectal cancer (RAPIDO): A randomised, open-label, phase 3 trial. Lancet Oncol..

[76] Ciseł B., Pietrzak L., Michalski W., Wyrwicz L., Rutkowski A., Kosakowska E., Cencelewicz A., Spałek M., Polkowski W., Jankiewicz M. (2019). Long-course preoperative chemoradiation versus 5 × 5 Gy and consolidation chemotherapy for clinical T4 and fixed clinical T3 rectal cancer: Long-term results of the randomized Polish II study. Ann. Oncol..

[77] Jin J., Tang Y., Hu C., Jiang L.-M., Jiang J., Li N., Liu W.-Y., Chen S.-L., Li S., Lu N.-N. (2022). Multicenter, Randomized, Phase III Trial of Short-Term Radiotherapy Plus Chemotherapy Versus Long-Term Chemoradiotherapy in Locally Advanced Rectal Cancer (STELLAR). J. Clin. Oncol..

[78] Conroy T., Bosset J.-F., Etienne P.-L., Rio E., François E., Mesgouez-Nebout N., Vendrely V., Artignan X., Bouché O., Gargot D. (2021). Neoadjuvant chemotherapy with FOLFIRINOX and preoperative chemoradiotherapy for patients with locally advanced rectal cancer (UNICANCER-PRODIGE 23): A multicentre, randomised, open-label, phase 3 trial. Lancet Oncol..

[79] Conroy T., Etienne P.-L., Rio E., Evesque L., Mesgouez-Nebout N., Vendrely V., Artignan X., Bouche O., Boileve A., Delaye M. (2023). Total neoadjuvant therapy with mFOLFIRINOX versus preoperative chemoradiation in patients with locally advanced rectal cancer: 7-year results of PRODIGE 23 phase III trial, a UNICANCER GI trial. J. Clin. Oncol..

[80] Fokas E., Allgäuer M., Polat B., Klautke G., Grabenbauer G.G., Fietkau R., Kuhnt T., Staib L., Brunner T., Grosu A.-L. (2019). Randomized Phase II Trial of Chemoradiotherapy Plus Induction or Consolidation Chemotherapy as Total Neoadjuvant Therapy for Locally Advanced Rectal Cancer: CAO/ARO/AIO-12. J. Clin. Oncol..

[81] Fokas E., Schlenska-Lange A., Polat B., Klautke G., Grabenbauer G.G., Fietkau R., Kuhnt T., Staib L., Brunner T., Grosu A.-L. (2021). Chemoradiotherapy Plus Induction or Consolidation Chemotherapy as Total Neoadjuvant Therapy for Patients with Locally Advanced Rectal Cancer: Long-term Results of the CAO/ARO/AIO-12 Randomized Clinical Trial. JAMA Oncol..

[82] Foulon V., Schöffski P., Wolter P. (2011). Patient adherence to oral anticancer drugs: An emerging issue in modern oncology. Acta Clin. Belg..

[83] Bosset J., Calais G., Daban A., Berger C., Radosevic-Jelic L., Maingon P., Bardet E., Pierart M., Briffaux A. (2004). Preoperative chemoradiotherapy versus preoperative radiotherapy in rectal cancer patients: Assessment of acute toxicity and treatment compliance: Report of the 22921 randomised trial conducted by the EORTC Radiotherapy Group. Eur. J. Cancer.

